# Supplementary figures and images for: Diversity and Host Interactions among Virulent and Temperate Baltic Sea Flavobacterium Phages
Source: Viruses. 2020 Jan 30;12(2):158. doi: 10.3390/v12020158 (PMC7077304; doi:10.3390/v12020158)

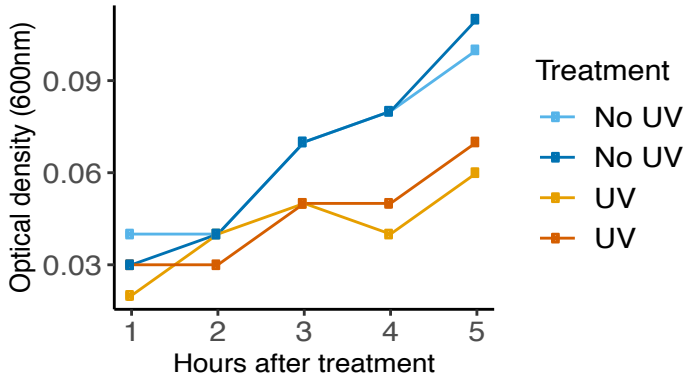

Figure S1. Optical density of LMO6 after the induction experiment with UVC.

Supplement: Supplementary file 1 [file viruses-12-00158-s001.zip › supplemental/S1_uv_od.pdf]
